# Representation of gender in migrant health studies – a systematic review of the social epidemiological literature

**DOI:** 10.1186/s12939-020-01289-y

**Published:** 2020-10-14

**Authors:** Lisa Wandschneider, Stephanie Batram-Zantvoort, Oliver Razum, Céline Miani

**Affiliations:** Department of Epidemiology and International Public Health, School of Public Health, Bielefeld University, POB 10 01 31, 33501 Bielefeld, Germany

**Keywords:** Gender, Migration, Social epidemiology, Systematic review, Gender equality, Structural determinants, Sexual orientation, Gender identity

## Abstract

**Background:**

Gender as a social construct contributes to determine who migrates and which migration-related risks and opportunities emerge in all phases of the migration trajectory. Simultaneously, migration influences the individual as well as societal definition and perception of gender roles. An explicit gender perspective in migration-related epidemiological research can contribute to adequately analyse and interpret the health of migrants. This systematic review gives a comprehensive overview on how gender has been conceptualised, operationalised and measured in social epidemiologic studies aiming to assess the influence of gender on health among migrants.

**Methods:**

We searched PubMed, Embase, CINAHL, the Cochrane Library, EconLit and PsycINFO and conducted backward reference searching. Reviewers independently selected studies, extracted data and conducted the quality assessment. Eligible studies actively aimed to understand, identify or explain the influence of gender on migrants’ health, whereby the role of gender can encompass a variety of mechanisms, processes or states of differentiation, discrimination and/or inequality.

**Results:**

Almost all of the 43 studies were cross-sectional and focussed on health outcomes in the post-migration phase. The most common theme of research was the health of male migrants in the US, and in particular of men who have sex with men (MSM). All studies treated gender as a binary variable (men vs. women), without discussing additional types of gender identities. A minority of studies differentiated clearly between sex and gender. Gender was mostly operationalised through attitudes toward gender roles and gender-based discrimination, experienced at the individual level. Community and societal level gender measures capturing structural gender determinants were underrepresented.

**Conclusions:**

The intersections of migration and gender suggested synergistic effects on health that only become visible when considering those two social determinants together. Future research needs to embrace a multilevel and non-binary understanding of gender and reflect on the influence of gender in the different phases of the migration journey.

**Systematic review registration:**

PROSPERO CRD42019124698.

## Background

### Sex, gender and health

Sex and gender are core determinants of health [1]. The term ‘sex’ describes the biological construct of anatomical, physiological, genetic and hormonal characteristics of human beings. In contrast to sex, gender refers to the socially constructed and context-specific characteristics of individuals. It encapsulates the prevailing norms, roles and relationships between and among individuals, which is highly context-specific and may vary by time and place [2].

The physiological sex and the social gender are interacting and depend on each other, not only in their meaning but also in their effect on health. Still, sex and gender are distinct concepts that need to be differentiated carefully, which represents a major challenge to health research [3–5]. Sex-linked biology influences for example the development of diseases (such as coronary heart disease) or the response to pharmaceuticals. Gender affects individual health-seeking or risk behaviour, environmental and occupational risks, health care utilisation and the compliance to treatment plans [6–8]. Within a broader context, sex and/or gender are linked to different levels of opportunity, control, access and influence. Thus, structural factors can promote or hinder individual access to care or health-promoting behaviours [9].

Over the past decades, it has been increasingly argued that the relevance and validity of health research could be enhanced by a more systematic consideration of the concept of gender [1, 9–11]. This is reflected in research funding schemes, publishing guidelines in scientific journals and good practice guidelines for epidemiology and public health [12–16]. In parallel, conceptual and methodological approaches have been developed to overcome the research gap on gender in public health and epidemiology [17–20]. There is now a consensus that reflecting on gender-related aspects in epidemiological research is not a “philosophical principle” but a dimension of research quality [1]. Gender-sensitive epidemiology has been promoted within the international and national research community [11, 21–23]. Its aim is to conceptualise and measure gender and its effect on health outcomes in order to reduce avoidable health inequalities related to gender.

Despite those advances, gender-sensitivity in research practice is still in its initial stages [21, 24, 25]. First, continuing not to take into account gender adequately as a health determinant (in addition to sex) may lead to gender bias in research, i.e. incomplete or biased evidence [26]. Gender bias impairs the validity and generalisability of findings leading to inadequate conclusions on for example risk factors, diseases and treatment [27, 28]. Second, the dominating dichotomisation and insufficient differentiation of sex and gender in epidemiology is reinforcing the traditional gender order. It prevents acknowledgement of gender and sexual diversity and fails to take into account health-related experiences that are of relevance to the society as a whole as well as to specific sub-populations such as genderqueer, intersex and trans persons [29]. Gender-based research should not reproduce marginalisation of any gender identity and needs to go beyond the conventional binary distinction of women and men [3]. Third, epidemiological studies identifying gendered differences often miss to critically assess and discuss the underlying mechanisms [30]. Gender theory that might help explain observed patterns is rarely applied [25].

### Gender, migration and health

Gender-sensitive approaches in epidemiology have become even more relevant with the increase of international migration. The so-called ‘super-diversity’ of modern societies challenges health care systems with regard to prevention, diagnosis and treatment of diseases, heterogenic exposures to behavioural and environmental risks, as well as the access to and use of health care services [31]. Among other factors, gender determines who migrates and which migration-related risks and opportunities emerge in all phases of the migration trajectory. Simultaneously, migration influences the individual as well as social definition and perception of gender roles [32, 33]. For example, women may experience their role differently when emigrating for economic reasons and going from principally caring for their family to the labour market. Homosexual and genderqueer persons may wish to escape from traditional gender norms (and gender-based discrimination) in their home country to settle in more gender-equal countries [34]. Gender norms describe social expectations and attitudes about how individuals should behave and act in a given society based on their sex assigned at birth. These effects apply in particular to international migration as differences in societal gender norms are likely to be more pronounced and complex between countries than within countries [35], which is why we refer to international migration in our review. Internalised gender norms and relations of the country of origin may be challenged and modified in the country of reception via acculturation processes and socioeconomic integration [32]. However, we acknowledge that cultural differences may in some contexts be more accentuated within countries, as a body of literature highlights [36, 37].

Compared to non-migrant populations, migrants tend to have on average a higher exposure to social risk factors, with challenging economic and social positions being related to occupational status, financial capacities, living situation and legal uncertainty [31, 38]. Compared to internal migration, the social mobility of international migrants is characterised by higher controls and regulations, e.g. with regards to access of basic civil rights such as education, employment, health care, political representation etc. [36]. Migrants also tend to have worse health status compared to natives, in particular with regard to mental health [31, 38, 39].

Gender and migration are mostly analysed separately in health research [24, 40]. This is in part due to the fact that both factors are complex and multidimensional, which makes an adequate and simultaneous representation of both concepts even more complicated [40]. Moreover, the data availability for migrant-specific as well as gender-sensitive research is limited, and it still remains difficult to do justice to the diversity of gender, especially in quantitative research [3, 41]. An explicit gender perspective in migration-related health research would contribute to adequately analyse, assess and interpret the health situation of migrants. Thus, increased efforts are needed to produce robust evidence on potential risk exposures and access to health care, on the basis of theoretical and empirical approaches [41].

### Research objectives

In this systematic review we addressed the research gap of gender-sensitivity in social epidemiology, at the intersection of health and migration. The general objective was to obtain a comprehensive overview on how gender has been conceptualised, operationalised and measured in social epidemiology focussing on the health status of migrants. More specifically, the review aimed to 1) understand which dimensions of gender are considered in epidemiological research on migrants’ health and at which level of analysis; 2) assess how gender is reflected on throughout the migration trajectory; and 3) identify good practice examples of gender-sensitive epidemiology in migrant populations.

## Methods

The review has been conducted and reported in adherence to the Preferred Reporting Items for Systematic Reviews and Meta-Analyses (PRISMA) (Supplementary file 1) [42].

### Searches

We designed our search strategy around three main concepts: migration, gender, and health. We used a modified PECO framework, which structures the research objectives of systematic reviews according to the four pillars of Population, Exposure, Comparator, and Outcome (PECO) [43]. In our review, migrants constitute the population of interest. The exposures are gender norms/roles/relations as well as gender-related discrimination and mechanisms producing gender inequality. The outcome is broadly defined as any health-relevant status following the WHO definition in the Ottawa Charta (1986) [44]. The comparison criterion is not applicable to this review. The search strategy was developed through consultation with experts in the fields of reviews and of gender-sensitive health research. After a pilot search in PubMed, we searched the following databases: CINAHL, PsycINFO, PubMed, the Cochrane Library, EconLit and Embase. (For search strategies applied to each database, see Supplementary file 2).

To reflect the heterogeneous migrant population, from economic migrants to displaced persons, we added terms referring to different subgroups, such as “undocumented”, “refugees” and “asylum seekers”. Additionally, we included ethnicity- and race-related terms, as these keywords are frequently applied in the US and the UK to describe migrant populations. However, the terms migrant, race, and ethnicity present distinct concepts. In epidemiology, race and ethnicity usually pertain to cultural and social characteristics, related to geographic origin, language and religious beliefs as well as biological characteristics, e.g. genetic origin or physical appearance [45]. In contrast, the classification of being a migrant is based on a life event or process, the migration here defined as one person moving from a country (country of origin) to another one (country of reception) to reside there [46]. For the purpose of this review, and in line with common practice in epidemiological research, we expand this definition to also include the direct offspring of immigrants. Including offspring of migrants who do not have a personal experience of migration is relevant because their legal, social and health-relevant living conditions might still be affected by the migration of their parents [45]. Thus, our study population of interest includes either people who migrated themselves or the offspring of immigrants (both parents migrated) [45].

The exposure category encompasses a variety of gender-related terms, referring to gender roles, inequality and discrimination. These terms derived from the literature on gender in the fields of sociology and public health, on activism websites and from conversations with gender experts. In addition to the gender related terms, we added search terms referring to sexual orientation, which describes one’s emotional, romantic or sexual attraction for other individuals. The distinction between gender and sexual orientation, and discrimination related to both concepts, is often not clear in the literature. For example, homophobia can be conceived as a gender-based discrimination as it stems from heteronormative assumptions on sexual orientation and gendered behaviour. We therefore included search terms for sexual orientation to ensure a comprehensive overview. Still, we aimed for a nuanced understanding of the concepts within this review and tried to disentangle them.

With regard to health outcomes, we adopted a broad definition to capture the diversity of gender-sensitive approaches within the whole spectrum of epidemiological research. For example, health-seeking behaviours, or being a victim of violence, were included as health outcomes.

In addition to the search in electronic databases, we conducted backward citation checks of full texts.

### Study inclusion and exclusion criteria

Studies were eligible for inclusion if they met the criteria described in Table 1.

**Table 1:** Eligibility criteria

Inclusion	Exclusion
1) Article actively aims to understand, identify or explain the influence of gender on health, whereby the role of gender can encompass a variety of mechanisms, processes or states of differentiation, discrimination and/or inequality 2) (At least one sub-group of) the study population meets the definition of migrants, understood as follows: individuals that either migrated themselves or whose both parents migrated 3) Epidemiological original research according to Porta (2008) [47], incl. Observational and interventional studies 4) Published before September 2019 and full text available (2nd screening stage) 5) No exclusion based on the language of the publication	1) Articles restricted to sex-specific health differences or analyses of the health of sexual minorities (i.e. lesbian, gay bisexual, trans, queer or intersex people -LGBTQI+) without considering the social dimension of gender (as opposed to sex or sexual dimension) 2) Gender norms/roles/relations are only considered in the discussion of findings and/or the underlying concepts and mechanisms are not operationalised in the data collection and/or analysis process 3) Migration is not a concept of major importance and migration-related characteristics are not explicitly analysed 4) Exclusively theoretical, methods development focussed, or policy-related research 5) Articles in form of editorials or commentaries 6) Qualitative study design

We only included studies reflecting on gender throughout the entire research process, i.e. in the background, study design, analysis and interpretation of findings. According to the current state of research, this is regarded as good practice in gender-sensitive epidemiology [14, 17, 19]. Given the relatively rich body of qualitative literature on gender norms [48] and the intersection of gender and migration [49–52], and the call for more theory-informed research in quantitative epidemiology integrating the social contexts, we decided to focus on quantitative epidemiologic research only to assess the status quo for this particular field.

In a pilot phase, the reviewers (LW, SBZ, CM) applied the eligibility criteria independently on a random sample of 5% of the studies. We then conducted the first screening phase at the level of titles and abstracts. In a second stage, we evaluated full-text articles for inclusion. At both stages, CM and SBZ each screened half of the records and LW screened all of them to double check the eligibility of studies. We resolved disagreements on the inclusion of full-text articles by discussions to achieve consensus, and if necessary, with the help of the third (and in exceptional cases a fourth) reviewer.

### Data extraction strategy

The data extraction comprised general study information as well as information about the integration of gender throughout the research process (see Supplementary file 3). Three reviewers (LW, SBZ and CM) independently conducted the extraction and compared data. In cases of missing information regarding the studies included, we contacted the corresponding authors by mail to obtain the missing information.

### Study quality assessment

In our original review protocol, we planned to use the Risk of Bias in Nonrandomized Studies – of Exposure (RoBINS-E) and of intervention (ROBINS-I). This, however, turned out to be not feasible since almost all the studies in this review were cross-sectional, and hence could not be assessed with the RoBINS-E/ROBINS-I [41, 42]. Instead, we used the National Heart, Lung, and Blood Institute (NHLBI) quality assessment tool for observational cohort and cross-sectional studies and their quality assessment tool for case control studies [53, 54], which have already been used for quality assessment in reviews [55, 56]. We conducted the quality assessment independently (CM and LW). Disagreements on the overall rating of the quality assessment were resolved by discussion to achieve consensus and if necessary, with the help of a third reviewer.

### Data synthesis and presentation

We synthesised the results in a narrative analysis and provided descriptive statistics about the main characteristics of the selected studies.

To map the identified gender measures, we adapted the multilevel gender analysis framework (GAF) by Jhpiego [57]. It was originally designed to identify “the evidence of gender inequalities relevant to programs focused on different levels of the health system” and to give guidance to health care staff on how to collect data in projects interested in gender-analysis. We maintained the core structure but adjusted it to our research question in order to help us understand how to operationalise gender, going beyond the sex-stratified questions in Jhpiego’s GAF. Thereby, the focus is no longer on health systems and interventions, but on gender measures categorised in levels and domains relevant to the health care system:

Levels (vertical scale):

- National level: gender-related indicators at the societal level
- Community, facility and institutional level (merged into one level in our analysis as we only identified two gender measures in the community and within health care facilities that did not allow any differentiation between the three levels).
- Individual and household level: e.g. subjective attitudes toward gender norms, roles and relations defined by the individual or within the household context, or personal experiences of discrimination without any specification of the context.

The domains (horizontal scale) characterise areas of social life and are originally based on work by the United States Agency for International Development’s (USAID) [58]:

- Access to assets: access to tangible and intangible resources, in form of capital, land, education, health care, political involvement etc.
- Beliefs and perceptions: expectations on how individuals should evolve in a specific society based on their gender or sex assigned at birth.
- Practices and participation: gendered behaviour indicating how social norms and beliefs are transferred into actions in social life (e.g. sharing household responsibilities, decision-making and power relations).
- Laws and policies: formal or informal rights or rules.

Just as in Jhpiego’s GAF, we understand the fifth domain, i.e. power, as an overarching result of (all) the four domains described above.

In the final step, we identified a good practice example of a gender-sensitive approach to migrants’ health in epidemiology. Based on recent standards of gender-sensitive social epidemiology and our research question, we defined the following indicators of good practice: (a) the study is theory-informed, actively seeking to understand the underlying mechanisms of gender effects on health [25, 59]; (b) the study clearly differentiates between gender and sex to reduce the risk for binary and stereotyped thinking [5, 60] –these first two indicators can be mutually supportive, as theoretical grounding helps to achieve conceptual clarity of the central gender concepts and thereby holds the potential for higher quality research [59]; (c) the study analyses the intersection of gender and migration; (d) the study is rated as “good” in the quality assessment.

As expected, we were not able to conduct a meta-analysis because of the large heterogeneity of exposures and outcomes.

## Results

**Fig. 1:**
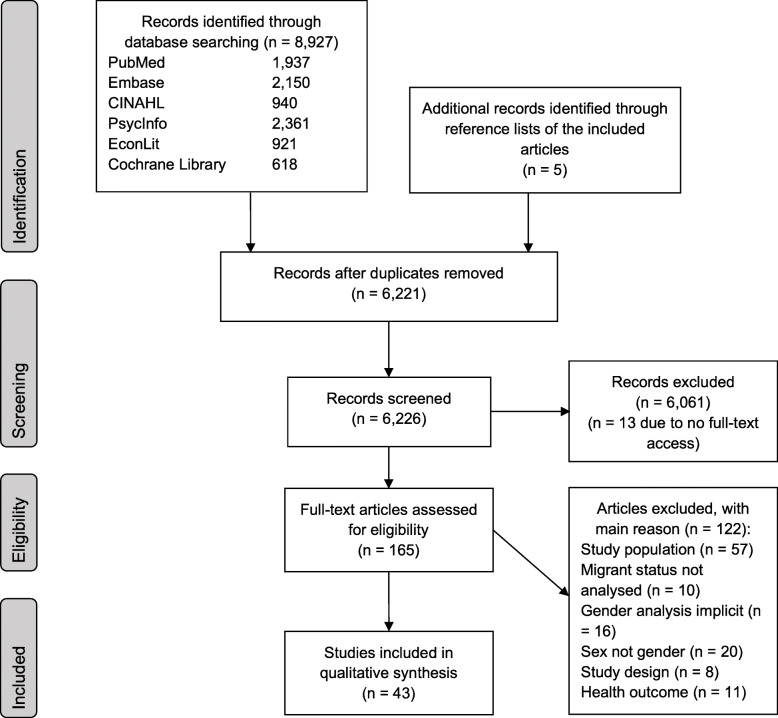
Flowchart of study selection process

We included 43 articles in the review (Fig. 1, supplementary file 3 – data extraction table). Of these, 39 studies had a cross-sectional research design [61–99], two were designed as cohort studies [100, 101] (although one conducted baseline analyses only), one study had a case-control design [102] and one study was a register-based secondary data analysis [103]. The sample size ranged from 62 [93] to 27,936 [101] participants, the median was 201 (mean = 342.3).

### Study population

Even though we searched for international epidemiological evidence (without regional limitations), the studies identified were restricted to Europe (*n* = 11) [61, 62, 64, 77, 78, 80, 86, 96, 100, 101, 103], Canada (*n* = 2) [68, 81] and the US (*n* = 31) [63, 65–67, 69–76, 79, 81–85, 87–95, 97–99, 102]. With regard to the origin of the migrant populations, we identified region-specific patterns of migration. In Europe, 7 out of 11 studies referred to migrants from undefined countries, which can mean, on the one hand, that they refer to a large number of countries of origin on a global level or that the countries of origin have not been specified [64, 77, 80, 86, 100, 101, 103]. The other European studies encompassed populations that migrated from Latin America (*n* = 2) [61, 62], Africa (n = 2) [61, 96] and the Dutch Antilles (*n* = 2) [78, 96]. In contrast, the US study populations were predominantly (first and second generation) migrants from Latin America (*n* = 20) [63, 65, 66, 70–75, 82–84, 87–92, 95, 97]. The second largest region of origin is Asia and the Pacific (*n* = 7) [69, 79, 81, 94, 98, 99, 102], one study referred to East and Western African migrants [93]. Only 3 studies in the US did not specify the region of origin [67, 76, 85].

Studies comparing migrants with native populations (*n* = 20) usually differentiated between native and foreign-born. Those only concerned with migrants (*n* = 23) mostly relied on the year of immigration [63, 66, 69–74, 76, 78, 79, 83, 84, 86, 87, 89–91, 93–95, 98, 102]. More differentiated analyses of the migration experience, e.g. by comparing first and second-generation migrants [69, 77, 79, 96], or taking into account mother tongue [66, 75, 82, 83, 92] and language competencies in the country of residence [63, 65, 70, 72, 75, 82, 83, 86, 88, 92], residence status [70, 71, 76, 92–94, 101] or reasons for migration [84, 86] were less frequent.

Nineteen out of 45 studies referred to gender and sexual minorities, understood as individuals who do not identify as heterosexual and/or cisgender [63, 65, 67, 68, 72, 73, 81–88, 90, 91, 93, 95, 99]. Of these, all but one (which focussed on LGB sexual minorities [81]) investigated the health of men –mostly men having sex with men, but also men self-identifying as gay, bisexual or transgender. Apart from two studies [81, 86], these were mostly conducted in the US.

One additional study recruited men without any reference to their sexual orientation [89]. In contrast to 20 studies solely focused on men’s health, we identified 8 studies on women only (none on gender and sexual minorities) [70, 71, 75, 76, 92, 101–103], which were again predominantly located in the US. The rest (*n* = 16) included men and women in their analyses [61, 62, 64, 66, 69, 74, 77–81, 94, 96–98, 100]. Those showed a more balanced distribution of study region with 8 out of 18 studies located in Europe [61, 62, 64, 77, 78, 80, 96, 100].

### Operationalisation of gender

The concept of gender was operationalised through the following dimensions: gender norms and roles (*n* = 22) [61, 62, 66, 69–75, 77–79, 81, 89–92, 94, 96–98, 102], gender-based discrimination (*n* = 17) [63, 65, 67, 68, 72, 76, 80, 82–88, 93, 95, 99], gender (in-)equality (*n* = 5) [64, 86, 100, 101, 103], gender-based violence (*n* = 3) [61, 77, 98] and gender relations (*n* = 2) [62, 75].

In 22 studies analysing gender roles and norms, we identified 20 gender measures in total, of which 6 measures assessed gender norms toward men (patriarchal beliefs, Machismo, notions of masculinity) [66, 70, 71, 74, 78, 79, 89, 91], 2 toward women (Chastity [79] or attitudes toward women [66, 69, 98]), 7 toward men and women (e.g. Bem-Sex-Role Inventory, sexism, double standards, sharing household responsibilities) [61, 62, 75, 92, 94, 96, 97], and 5 towards homosexuality [72, 81, 90, 91]. Gender-based discrimination was almost exclusively related to sexual orientation, measuring experiences and internalisation of homosexual or transsexual stigma [63, 65, 67, 68, 72, 76, 80, 82–88, 93, 95, 99]. Two studies assessed discrimination based on sex assigned at birth [76, 80].

Gender (in-)equality was operationalised either with the help of an index of female social, political, and economic participation at the national level provided by the United Nations (Gender Empowerment Measure [64], Gender Equity Index [103], Gender Inequality Index [100]), the World Economic Forum (Global Index Gap [101]) or by national laws promoting unequal treatment of sexual minorities (rainbow map of the International lesbian, gay, bisexual, trans and intersex association (ILGA) [86]). These gender measures were all built on national statistics of, for example, the labour market, in contrast to all other dimensions of gender that were self-reported.

Three measures explored gender-based violence: attitudes supporting intimate partner violence (IPV -defined as emotional, physical, sexual and economic violence perpetrated by an intimate partner) [98], the normalisation of gender-based violence (as a subscale of sexism) [61] and violence legitimising norms of masculinity [77]. Gender relations were operationalised as the power and control of decision-making in a relationship [62, 75].

The adapted GAF mapping all operationalisations of gender illustrates that the vast majority (84%) is conceptualised at the individual level, with the domain beliefs and perceptions accounting for the largest proportion (*n* = 24, 56% of all gender operationalisations) and the domain practices and participation for the second largest (*n* = 12, 28% respectively). At the community level, we identified two operationalisations (both in the same study) in the domains of beliefs and perceptions and practices and participation, the other domains were not represented. The societal level was analysed through 5 measures in the domains access to assets (*n* = 4) and policies and laws (*n* = 1) (Table 2).

**Table 2:** Gender analysis framework

		Domains			
		Access to assets	Beliefs and perceptions	Practices and participation	Laws and policies
**Level of analysis**	**Societal level**	**Gender (In-)Equality Scores** Gender Empowerment Measure [64] Gender Inequality Index [100] Global Gender Gap [101] Gender Equity Index [103]			ILGA rainbow map [86]
	**Community, facility, institutional level**		Sexual minority structural stigma [85]	Sexual minority structural discrimination [85]	
	**Individual level**		**Attitudes towards Homosexuality** Reactions to Homosexuality Scale [72, 73, 91], Internalized negative attitudes about gay men and MSM [90], Homophobia [91], Attitudes toward Lesbian and Gay Men [81], Index of Attitudes toward Homosexuals [81], Double Standard Scale (sexuality) [62], Sexism [61] **Gender norms** Adherence to traditional notions of masculinity [91], Adherence to traditional masculine norms [89], Idealised Beliefs about Masculinity Scale [79], Patriarchal ideology [74], Machismo [66, 78], Marianismo Beliefs Scale [70, 71], Chastity scale [79], Attitude Toward Women Scale [66, 69, 98, 102], Bem-Sex-Role Inventory [92, 97], Gender ideology binary item [94], Gender-role beliefs [96], Gender-role index [75] **Discrimination of sexual orientation** Internalised homophobia [65, 68, 88, 93], Openness about one’s same-sex attraction in the country of origin [93], Reasons for discrimination: sex, sexual orientation, transphobia, pregnancy [80], **Attitudes toward gender-based violence** Violence legitimizing norms of masculinity [77], Intimate partner violence supporting attitudes [98]	**Gendered behaviour** Sharing household responsibilities [96], Gender power relations [75], Sexual Relationship Power Scale [62] **Discrimination of sexual orientation** Conversations about discrimination with family and friends [99], Homophobic violence in the country of origin [93], Hiding sexual orientation in country of residence [93], Experienced homosexual stigma (by Diaz 2001) [63, 68, 72, 87, 99], Experiences of homophobia [65, 67, 82, 83, 88], Perceived discrimination of sexual orientation [95], Gender discrimination measured as part of the Cumulative Trauma Scale-Short [76], Sexual migration [84] **Gender-based violence** Normalisation of gender-based violence (as a dimension of sexism) [61]	

Abbreviation: *ILGA* International lesbian, gay, bisexual, trans and intersex association

### Gender migration interaction

In 35 of 43 studies, gender was conceptualised in the post-migration phase and referred to norms or experiences related to the country of reception [61–75, 77–83, 87–92, 94–99, 102]. Five studies used gender concepts with regard to the pre-migration phase, operationalised as the motivation to migrate due to discrimination based on sexual orientation [84], gender inequality [100, 101, 103] or the structural stigma against sexual minorities experienced in their hometown [85] in the country of origin. In two studies pre- and post-migration gender measures were operationalised by comparing anti-gay stigma [86] and openness about one’s sexual orientation [93] in the country of origin and reception. Kira et al. assessed gender discrimination with the help of the Cumulative Trauma Scale that refers to trauma acquired throughout the migration process, but it does not allow a migration-phase specific analysis [76].

In addition to the migration phase specific conceptualisation of gender, less than half of the studies analysed interactions between gender and migration variables (*n* = 20) [61, 62, 65, 69, 75, 77, 81–86, 88, 92, 93, 96, 98, 100, 101, 103]. In these studies, we identified five different strategies analysing how gender and migration can affect each other, i.e. interact. First, this was operationalised as a variable containing information on gender and migration. For example, by combining homophobia and racism in a social discrimination index, Mizuno et al. showed that multiplicative effects explain health disparities [82, 83]. Other operationalisations included migration to affirm sexual orientation more openly and to avoid persecution [84], the gender equality scores [100, 101, 103] as well as the openness about sexual orientation [93] in the country of origin and reception. Second, stratified analyses by migrant status were conducted to compare the gender-related norms in different subgroups. However, the results of the stratifications were inconsistent. While four studies found generational differences in gender-role beliefs with first-generation migrants showing stronger beliefs [61, 77, 81, 96], three other studies did not [69, 75, 88]. Third, pathway analyses were applied to identify direct and indirect effect of gender and migration (or vice versa) on health. In their analysis, Bruce et al. found that foreign-born have higher levels of internalised homosexual stigma, which is negatively associated with substance use and sexual risk behaviour [65]. Yoshihama et al. identified an indirect effect of enculturation on IPV supporting attitudes through the association with patriarchal gender norms [98]. Fourth, an interaction term was added in a regression analysis. Pachankis et al. thereby showed that the association of anti-gay stigma and HIV prevention outcomes was stronger for MSM migrants living in receiving countries with high anti-immigrant stigma [86]. Fifth, interaction analyses were conducted through bivariate analyses in three studies [62, 85, 92].

### Gender effects on health outcomes

The most prevalent health outcomes were mental health (*n* = 15) and sexual health (*n* = 15). Mental health outcomes were depression [66, 67, 79, 85, 99] or depressive symptoms [81, 90], anxiety [67, 69, 85], stress [71, 77, 81, 94], trauma [76, 92] and psychological well-being [87, 96]. Sexual health was investigated in studies about gender and sexual minorities (except for three studies [62, 70, 78]). It was operationalised through sexual risk behaviour, i.e. unprotected intercourse and the resulting risk for sexually transmitted infections (STI) [62, 65, 70, 82, 83, 85, 88, 93, 99], engagement in transactional sex [93] or sex under the influence of alcohol and/or drugs [65, 70] and condom use [78, 91]. Additionally, HIV specific outcomes were assessed, such as, HIV prevention actions and motivations [86, 93], HIV testing behaviour [73, 95] and HIV status [84].

Health determinants (*n* = 11) included a variety of variables ranging from alcohol [64, 72, 82, 83, 85, 97] and drug use [82, 83, 85], aggression [100], discrimination experiences [80] to breastfeeding [101], birth control practices [74] and health promoting behaviour [63]. Gender-based violence was assessed in 5 studies and captured attitudes towards IPV [74, 98] as well as reported acts of IPV [61, 75, 102]. Fernbrant et al. examined the premature death due to external causes and interpersonal violence [103]. Three studies assessed the access to care, looking at urgent vs. non-urgent care [95] and the use of health [89] and mental health services [68].

The qualitative synthesis of the gender effects on health outcomes indicated as expected that stronger adherence to traditional gender norms [62, 66, 70, 71, 77, 78, 81, 92, 94, 102], higher levels of gender inequality [64, 100, 103], gender-based discrimination [63, 65, 67, 76, 82–88, 91, 93, 95] or gender-based violence [61, 77, 98] are associated with adverse health outcomes. Four studies showed that gender effects only manifested in interaction with other variables. One of these interacting variables was the migrant status operationalised as generational status, i.e. gender effects were only significant in first-generation migrants [69]. Mizuno et al. demonstrated that homophobia or racism alone did not affect sexual risk behaviour, but exposure to both homophobia and racism was significantly associated with increased odds of sexual risk [82, 83]. Moreover, this association was only significant among foreign-born but not US-born men (in spite of almost identical proportions of discrimination in the two groups) [83]. In the third study, Vanderlinden et al. found a composition effect of gender inequality and maternal education (measured with the International Standard Classification of Education). Gender inequality was only significantly associated with higher chances of exclusive breastfeeding when the mother’s education was added to the model [101].

However, some studies did not show any effect of gender measures on health [72–74, 79, 90, 99]. Others suggested that stronger beliefs in traditional gender norms or originating from countries with lower scores of gender equality can have protective effects on health, e.g. by reducing alcohol use [97] or by increasing the uptake of health care services [89] and exclusive breastfeeding [101]. Yet, these findings represented only a minority in the overall synthesis of findings.

### Study quality assessment

As a result of the QA process (more details in Supplementary file 4), we rated 16 studies as Good [64, 66, 75, 77, 78, 80, 86, 89, 92, 94, 96–98, 100, 101, 103], 24 as Fair [61–63, 65, 67, 68, 70–73, 76, 81–85, 87, 88, 90, 91, 93, 95, 99, 102] and 3 as Poor [69, 74, 79] The three studies that were considered of poor quality were lacking details with regard to how the exposure(s) or outcome(s) were measured, to the exclusion and inclusion criteria applied to the participants and to the recruitment process.

Only a minority of the studies relied on probability sampling. All others relied on non-probability sampling, e.g. respondent driven sampling or convenience sampling, because most of the studies defined their populations as “hard-to-reach”. The authors did not strive for representative samples, and their analyses were rather exploratory in nature. Accordingly, the lack of representative samples was not rated as a major flaw.

Most of the gender measures were self-reported and subjective, but again this is due to the nature of the dimensions assessed (discrimination, gender roles) and does not necessarily present a major flaw in the measurement. Most of the studies included statistical analyses that were appropriate and considered relevant confounders. With regard to reporting standards, many studies failed to give details on the selection criteria, response rates and the time periods covered.

### Reflections on a good practice example

Based on the indicators introduced before (see Methods section), we have identified one article as example of good practice. Although the authors applied a binary understanding of gender (like all studies in our review), Nivette et al. scored highest on our set of indicators [100]. They investigated sex differences in aggressive behaviour among school-aged children residing in Zurich, Switzerland,

Nivette’s article was the only theory-driven study actively aiming to examine and compare theoretical assumptions [100]. Two contradicting theoretical perspectives, the social role theory and sexual selection theory, were guiding the research. While social role theory assumes that socialisation and gender roles form sex differences in aggression, sexual selection theory postulates that variation in ecological contexts trigger biological factors affecting sex differences. These theoretical perspectives defined the research question, hypotheses and statistical tests but also provided the basis for the background and discussion. Accordingly, the whole research process was directed towards the verification of the theoretical perspectives.

Eight other studies also drew on theoretical frameworks, but their approach was different to the one Nivette et al. followed. These were used to define a theoretical perspective that either emphasised the public health relevance of the empirical question, justified the selection of variables or different levels of analysis included in statistical models, or guided the statistical methods [63, 65, 70, 71, 79, 87, 88, 95]. Theoretical or conceptual frameworks included for example Bronfenbrenner’s social ecological theory [70, 71], intersectionality [79], Latkin’s dynamic social systems framework [95], minority stress framework [65] and Diaz’s psycho-cultural model [63].

Conceptual clarity on the terms sex and gender was achieved in 4 articles including Nivette et al. [100]. However, those three other studies either lacked theoretical grounding completely [61, 75] or referred to but did not apply a theoretical framework [101]. In our good practice example, the theoretical grounding helped clearly delineate the influence of sex and gender and adequately operationalise the social concept of gender.

In 13 studies, sex and gender were used interchangeably or gender was used to refer to sex [62, 64, 66, 74, 78–80, 94–98, 103]. For the other 26 studies it was not possible to determine whether the authors achieved conceptual clarity because either the terms gender and/or sex had not been applied (mostly in studies concerned with men or women only) [63, 67–71, 76, 77, 81, 83, 84, 87, 92, 93, 99, 102] or the term gender exclusively referred to gender and sexual minorities [65, 72, 73, 82, 85, 86, 88–91].

The interaction of gender and migration-related variables was the primary interest for five studies (of 19 studies in total investigating the interaction, see respective results section). Nivette et al. used the gender inequality index of the parents’ country of birth and thereby determined the gender polarisation in the parent’s society of origin [100]. Four other studies should be highlighted here as well, because their primary interest was the interaction of gender- and migration-related variables. Pachankis et al. (2017) and both studies of Mizuno et al. suggested that homophobic and anti-immigrant discrimination interact and thereby affect health outcomes differently compared to additive analyses [82, 83, 86]. The other article by Pachankis et al. (2016) focused on experiences throughout the migration trajectory, i.e. experiences upon arrival and hometown characteristics, and compared the motivations for sexual migration for each of these characteristics [85].

As the GAF highlighted, few studies focused on the societal level and considered structural determinants of health [64, 85, 86, 100, 101, 103]. Nivette et al. did it in two ways: using a macro-level indicator (Gender Inequality Index-GII) to measure gender inequality in the country of origin, but also referring to potential structural determinants of inequality in the country of reception when they insisted on the fact that their population lived in an affluent environment with low unemployment, good access to child- and healthcare and where ecological variation was minimized [100]. Similarly, Pachankis et al. explicitly identify MSM migrants as an intersectional population facing structural stigma directed toward their sexual orientation and toward being a migrant [86]. Vanderlinden et al. emphasised that gender inequality is not just a question of legislation and social policy measures, but of how these norms need to be incorporated into cultural traditions, be it in the country of origin or reception [101].

Finally, a “good” rating in the quality assessment was considered a criterion of good practice to minimise the risk of biased results. Next to Nivette et al., 15 other studies achieved a good overall rating [64, 66, 75, 77, 78, 80, 86, 89, 92, 94, 96–98, 100, 101, 103]. However, these did not meet the other good practice criteria. Half of those examined interactions between gender and migration [75, 77, 86, 92, 96, 98, 101, 103], only two achieved conceptual clarity between sex and gender [75, 101] and none of them were theory-informed.

## Discussion

Gender in epidemiological studies on migrant health was mostly operationalised through gender norms and gender-based discrimination, experienced at the individual level. The intersections of migration and gender suggested synergistic effects on health that only become visible when considering both social determinants concomitantly.

As expected, the findings indicated that stronger adherence to traditional gender norms, higher levels of gender inequality, gender-based discrimination, and gender-based violence were associated with adverse health outcomes. The health outcomes reflected the common focus of gender-related research on mental and sexual health as well as gender-based violence [104]. The evidence was dominated by research on gender and sexual minorities which is reflection of the fact that sex, sexuality and gender are very difficult to disentangle. Research focussing on LGBTQI+ participants was almost exclusively restricted to the US. Most European and Canadian studies included both men and women, mostly heterosexual and cisgender. No study was specifically about transgender persons or lesbian women.

We identified the following gaps: All studies treated gender as a binary variable (men vs. women) and did not try to conceptualise or discuss additional types of gender identities. This has already been criticised in previous analyses [5, 104]. In spite of the theoretical and conceptual developments that go beyond a binary understanding of gender [59, 105], there still seem to be empirical barriers to meeting those requirements. Recent developments in quantitative survey methods, e.g. suggesting a transgender-inclusive sex/gender measure [29], may improve the status quo in future research.

The GAF revealed a strong focus on individual behaviours and risk factors, and a clear lack of community and societal level gender measures to capture structural gender determinants. This insufficient integration of macro determinants is often criticised in social epidemiology [106, 107]. Based on our findings, we are reiterating the call for an increased attention of the macro level for the field of gender and migration in social epidemiology [108, 109]. If we want to understand external, concrete sources of discrimination and disadvantage in the lived experiences of individuals, we must also know where they come from and how they are reinforced or mitigated at the community level. The same applies to the societal level. In addition, it should be taken into account that the different levels are mutually interdependent, and that national level policies and community services could lead to greater gender equality at the household level (e.g. paid parental leave and availability of child-care services) [109]. Besides the limited presence of community and societal level measures on a vertical scale, our review also indicated limited range at a horizontal scale, as gender was mostly operationalised as beliefs and perceptions. However, epidemiological research must acknowledge that gender is not restricted to norm systems but is also expressed through every-day experiences and actions in terms of access to assets, practices and participations and formal and informal rights. Accordingly, future research should integrate more dimensions of gender to allow for multi-level and multi-dimensional analyses of gendered pathways to health, despite the methodological challenges that arise from analysing community and societal-level determinants [104, 106].

Just as in global survey data [104], none of the studies analysed who or which institutions enforce gender norms and how. However, the review points out how non-conforming behaviours in heteronormative systems can be associated with discrimination, poor mental health status and higher subjective barriers to seek health care services. Evidence on how gender norms are created and reinforced, be it within the general population, specific subgroups such as migrant populations, or within the health care system, is crucial for designing effective gender-transformative policies and interventions reducing health inequalities.

Most of the research was set in the US, which explains the focus on Latinx immigrants (Latinx is used in place of Latino/Latina to include men, women and non-binary persons of Latin American origin). Few studies were located in Europe, even fewer in Canada. This is a common finding in both research fields, i.e. migration research as well gender-sensitive public health [110, 111]. Gender-related factors were mostly analysed in the post-migration phase. One possible explanation for this is the general lack of data on pre-migration phases and the lack for longitudinal health studies encompassing the migration trajectory. If we want to enhance a context specific, fluid social understanding of gender, the migration experience in its entirety should be considered. This would require, on the one hand, longitudinal studies on migrants’ health that include rich gender-related data as well as data on the migration trajectory, and, on the other hand, sufficient sample sizes and power to allow for in-depth analysis of intersections of gender and migration (and other social determinants) and their impact on health [104].

In addition to the gaps in the literature, our reflection on good practices identified some examples of how gender and migration should be analysed together.

Besides confirming that theory-informed research is scarce in epidemiology and quantitative health research on gender [25, 112, 113], the review allowed to compare different strategies of how to integrate theory. Theory use was often limited to either selecting influencing factors, testing hypotheses, integrating theoretical considerations in the background or to interpret the empirical findings. Our good practice example integrated gender theories in all phases of the research process and aimed to illustrate the validity of two contradicting theories with the aim to better understand health behaviour. This again points out the importance of theory in gender-sensitive epidemiology.

It is striking that conceptual clarity on the terms sex and gender was implemented only in few studies, and not even in studies with an explicit focus on a social understanding of gender. This is consistent with previous analyses [5]. Our reflection on good practice characteristics highlighted that a theory-informed approach does not only imply conceptual clarity of the terms sex and gender but also allows to distinguish between social and biological effects on health in an empirical analysis. The conceptual and theoretical clarity thus achieved holds the potential for more nuanced and higher quality research in gender-sensitive epidemiology and addresses the current need for a contextualisation of research questions, justification of theoretical standpoints and the appropriate use of the selected concepts [59].

Moreover, the review suggested different strategies for statistical analysis and the assessment of the intersection of gender and migration. Most of the studies used stratified subgroup comparisons or interaction terms in regression analyses, but some also examined indirect and direct effects in pathway analyses. To explore the multiple levels of determinants of health inequalities, multilevel analyses have gained popularity in quantitative public health studies and social epidemiology [114, 115]. In recent years, intersectionality-based methods for quantitative health research started to emerge that are designed to take into account the complex interplay of multiple social determinants shaping health inequalities and which could be of interest for future studies in the field of gender and health of migrants [10, 25, 116]. In addition to taking into account migration, some studies go even further and tried to show how other social determinants can intersect with gender and/or migration and thereby impact health, reiterating the call for intersectionality-based analyses [10, 117]. Within the intersectionality framework, Merlo et al. developed an enhanced version of a multilevel approach with their so-called multilevel analysis of individual heterogeneity and discriminatory accuracy (MAIHDA), thereby providing a powerful and theory-informed quantitative approach to model health inequalities across intersecting social positions [118, 119].

### Strengths and limitations

To our knowledge, this is the first systematic review assessing how gender as a social construct is represented in epidemiological migration-related research. Our broad understanding of “gender” encompasses various levels of analysis, dimensions and topics. Mapping the operationalisations of gender within the GAF strengthens the gender analysis for health outcomes and makes our findings directly comparable to previous research. The comprehensive search strategy and the multiple databases ensured a broad coverage of international epidemiological evidence published in academic journals. The definition for gender and the explicit distinction from ‘sex’ and ‘sexual orientation’ we applied allowed a nuanced analysis of the current gender-sensitive research practice and its’ intersection with migration. Moreover, it highlighted that gender-sensitive research can and must go beyond sex-stratified analyses and provides in-depth understanding of gender and its’ synergistic effects with migration and other social determinants.

Some limitations have to be noted: the review mostly relied on cross-sectional studies with a wide range in the sample size and heterogenous measures on gender, migration and health outcomes. This makes it difficult to compare the study results and to identify clear gender effects on the health of migrants. Associations identified do not determine the timing of exposure and outcome as all data are gathered simultaneously. The estimates, especially in the studies with small sample sizes, might overestimate the effects of gender and migration on the respective health outcomes due to low statistical power and have, in any case, limited generalisability. In addition, our search strategy might have missed studies on Latinx (or other ethnic minorities) immigrant populations that were not explicitly named as such, and studies that discussed gender-relevant aspects without using the term or other related search terms. However, we still identified many studies on Latinx as well as studies that did not use the term “gender”, so we believe that the number of missed articles would be small and would not affect the findings of this review.

### Implications on equity in health

From a methodological perspective, our review has the following implications for social epidemiological research on migrants and/or people who experience disadvantages because of their gender. First, to improve equity in health for disadvantaged groups, it is crucial to adequately understand the mechanisms at play in creating disadvantage and imbalance of power. Yet, the identification of such mechanisms is impeded, when the methodological approach in epidemiological studies is not capturing the entirety of experiences linked to gender and migration. This review shows that the literature is only starting to take into account the structural dimension of gender which makes up for an important part of the gender experience in people’s life. The same applies to the dimension of migration, as most of the studies assess the health of migrants from an acculturation perspective and thereby place the burden of poor health statuses on individuals. Emphasising the need to investigate the structural dimension moves the responsibility of better health from the (disadvantaged) individuals to the (oppressing) system-level actors [120–122]. It is a renewed call for action, guided by the principles of equity in health and social justice [108, 123].

Second, our findings indicate that the framing of socially constructed groups as homogenous (as it is often the case for migrants, women, MSM, LGBTQI+ and many others) is inadequate, because people within these groups may experience different health outcomes along the intersectional axes of inequalities [118, 124]. Recognising the intersectional positions in populations will allow more effective, targeted health programmes and public health policies aiming to reduce health inequalities.

## Conclusion

Gender in epidemiological studies on migrant health was mostly measured in the post migration phase and at the subjective, individual level. Structural characteristics at the community and societal level as well as earlier phases of the migration trajectory remained underrepresented. The intersections of gender and migration indicated that gender is understood as a context-specific, social construct and that gender and migration interactions might have multiplicative effects on health outcomes that are invisible when investigated separately. More research is needed on populations outside the US, on migrant women, and on the influence of gender on the different phases of the migration journey. Methodological challenges to be addressed in future research include: 1) embracing a multilevel and non-binary understanding of gender, 2) aiming for a careful application of sex and gender terms, 3) increasing theory-informed analyses to understand how gendered pathways affect health and 4) improving data availability for both gender and migrant-sensitive analyses.

## Supplementary information

**Additional file 1.**

**Additional file 2.**

**Additional file 3.**

**Additional file 4.**

## Data Availability

Not applicable.

## Abbreviations

GAF: Gender assessment framework
ILGA: International lesbian, gay, bisexual, trans and intersex association
LGBTQI+: Lesbian, gay bisexual, trans, queer or intersex people
IPV: Intimate partner violence
MSM: Men having sex with men
NHLIB: The national heart, lung, and blood of the national institutes of health
PRISMA: Preferred reporting items for systematic reviews and meta-analyses
US AIDS: United States agency for international development
