# Supplementary material for: Representation of gender in migrant health studies – a systematic review of the social epidemiological literature
Source: Int J Equity Health. 2020 Oct 14;19:181. doi: 10.1186/s12939-020-01289-y (PMC7556985; doi:10.1186/s12939-020-01289-y)
Supplement: Supplementary file 2 — Additional file 2. [file 12939_2020_1289_MOESM2_ESM.docx]

# Search strategies

All searches were conducted on the 20^th^ February 2019.

## PubMed (1,937)

("migration" OR "migrant" OR "migrants" OR "immigrant" OR "immigrants" OR “refugee” OR “refugees” OR “asylum” OR “undocumented” OR "first generation" OR "second generation" OR "ethnic minorities" OR "ethnic minority" OR “racial minority” OR “racial minorities” OR "refugees"[MeSH Terms] OR ("emigration and immigration"[MeSH Terms])

AND (“health” OR “well-being” OR “mortality” OR "life expectancy" OR “morbidity” OR “risk” OR “disease” OR "physical" OR "psychological" OR "mental health" OR "health inequalities" OR "depression" OR “anxiety” OR “psychosis” OR “stress” OR “minority stress” OR “PTSD” OR “post-traumatic stress syndrome” OR health status OR health equity OR health service OR sexual health OR child’s health OR women’s health OR men’s health OR health behaviour OR health promotion OR health information)

AND (“gender”[Title] OR “gender difference” OR “gender differences” OR “gender-based” OR “gender-dependent” OR “gender violence” OR “gender bias” OR “sex bias” OR “gender relations” OR “gendered violence” OR “gender discrimination” OR “gendered discrimination” OR “gender inequalities” OR “gender inequality” OR “gender equality” OR “gender identity” OR “gender expression” OR “gender presentation” OR “gender normative” OR “gender non-conforming” OR “gender role” OR “gender norm” OR “gender relation” OR “masculinity” OR “feminity” OR “feminine presenting” OR “masculine presenting” OR “doing gender” OR “gender mainstreaming” OR “domestic violence” OR “sexism” OR ”racialised” OR “racialized” OR “homophobic” OR “transphobic” OR “homophobe” OR “transphobe” OR “homophobia” OR “transphobia” OR “sexist” OR “misogyny” OR “heteronormativity” OR “LGBT” OR “LGBTQI” OR “transgender” OR “transgendered” OR “transsexual” OR “cisgender” OR “intersex” OR “sexual minority” OR “sexual minorities” OR “gender minorities” OR “gender minority” OR “queer” OR “gay” OR “lesbian” OR “sexual orientation” OR “Men Who Have Sex With Men” OR “Women Who Have Sex With Women“) Filters: Human

"migration"[All Fields] OR "migrant"[All Fields] OR "migrants"[All Fields] OR "immigrant"[All Fields] OR "immigrants"[All Fields] OR "refugee"[All Fields] OR "refugees"[All Fields] OR "asylum"[All Fields] OR "undocumented"[All Fields] OR "first generation"[All Fields] OR "second generation"[All Fields] OR "ethnic minorities"[All Fields] OR "ethnic minority"[All Fields] OR "racial minority"[All Fields] OR "racial minorities"[All Fields] OR "refugees"[MeSH Terms] OR "emigration and immigration"[MeSH Terms]

AND ("health"[All Fields] OR "well-being"[All Fields] OR "mortality"[All Fields] OR "life expectancy"[All Fields] OR "morbidity"[All Fields] OR "risk"[All Fields] OR "disease"[All Fields] OR "physical"[All Fields] OR "psychological"[All Fields] OR "mental health"[All Fields] OR "health inequalities"[All Fields] OR "depression"[All Fields] OR "anxiety"[All Fields] OR "psychosis"[All Fields] OR "stress"[All Fields] OR "minority stress"[All Fields] OR "PTSD"[All Fields] OR "post-traumatic stress syndrome"[All Fields] OR ("health status"[MeSH Terms] OR ("health"[All Fields] AND "status"[All Fields]) OR "health status"[All Fields]) OR ("health equity"[MeSH Terms] OR ("health"[All Fields] AND "equity"[All Fields]) OR "health equity"[All Fields]) OR ("health services"[MeSH Terms] OR ("health"[All Fields] AND "services"[All Fields]) OR "health services"[All Fields] OR ("health"[All Fields] AND "service"[All Fields]) OR "health service"[All Fields]) OR ("sexual health"[MeSH Terms] OR ("sexual"[All Fields] AND "health"[All Fields]) OR "sexual health"[All Fields]) OR (child's[All Fields] AND ("health"[MeSH Terms] OR "health"[All Fields])) OR ("women's health"[MeSH Terms] OR ("women's"[All Fields] AND "health"[All Fields]) OR "women's health"[All Fields]) OR ("men's health"[MeSH Terms] OR ("men's"[All Fields] AND "health"[All Fields]) OR "men's health"[All Fields]) OR ("health behaviour"[All Fields] OR "health behavior"[MeSH Terms] OR ("health"[All Fields] AND "behavior"[All Fields]) OR "health behavior"[All Fields]) OR ("health promotion"[MeSH Terms] OR ("health"[All Fields] AND "promotion"[All Fields]) OR "health promotion"[All Fields]) OR (("health"[MeSH Terms] OR "health"[All Fields]) AND ("Information (Basel)"[Journal] OR "information"[All Fields])))

AND ("gender"[Title] OR "gender difference"[All Fields] OR "gender differences"[All Fields] OR "gender-based"[All Fields] OR "gender-dependent"[All Fields] OR "gender violence"[All Fields] OR "gender bias"[All Fields] OR "sex bias"[All Fields] OR "gender relations"[All Fields] OR "gendered violence"[All Fields] OR "gender discrimination"[All Fields] OR "gendered discrimination"[All Fields] OR "gender inequalities"[All Fields] OR "gender inequality"[All Fields] OR "gender equality"[All Fields] OR "gender identity"[All Fields] OR "gender expression"[All Fields] OR "gender presentation"[All Fields] OR "gender normative"[All Fields] OR "gender non-conforming"[All Fields] OR "gender role"[All Fields] OR "gender norm"[All Fields] OR "gender relation"[All Fields] OR "masculinity"[All Fields] OR "feminity"[All Fields] OR (feminine[All Fields] AND presenting[All Fields]) OR "masculine presenting"[All Fields] OR "doing gender"[All Fields] OR "gender mainstreaming"[All Fields] OR "domestic violence"[All Fields] OR "sexism"[All Fields] OR "racialised"[All Fields] OR "racialized"[All Fields] OR "homophobic"[All Fields] OR "transphobic"[All Fields] OR "homophobe"[All Fields] OR "homophobia"[All Fields] OR "transphobia"[All Fields] OR "sexist"[All Fields] OR "misogyny"[All Fields] OR "heteronormativity"[All Fields] OR "LGBT"[All Fields] OR "LGBTQI"[All Fields] OR "transgender"[All Fields] OR "transgendered"[All Fields] OR "transsexual"[All Fields] OR "cisgender"[All Fields] OR "intersex"[All Fields] OR "sexual minority"[All Fields] OR "sexual minorities"[All Fields] OR "gender minorities"[All Fields] OR "gender minority"[All Fields] OR "queer"[All Fields] OR "gay"[All Fields] OR "lesbian"[All Fields] OR "sexual orientation"[All Fields] OR "Men Who Have Sex With Men"[All Fields] OR "Women Who Have Sex With Women"[All Fields]) Filter: Humans

## Embase (2,150)

('migration' OR 'migrant' OR 'migrants' OR 'immigrant' OR 'immigrants' OR 'refugee' OR 'refugees' OR 'asylum' OR 'undocumented' OR 'first generation' OR 'second generation' OR 'ethnic minorities' OR 'ethnic minority' OR 'racial minority' OR 'racial minorities' OR 'refugee'/exp OR 'immigration'/exp)

AND ('gender':ti OR 'gender difference' OR 'gender differences' OR 'gender-based' OR 'gender-dependent' OR 'gender violence' OR 'gender bias' OR 'sex bias' OR 'gender relations' OR 'gendered violence' OR 'gender discrimination' OR 'gendered discrimination' OR 'gender inequalities' OR 'gender inequality' OR 'gender equality' OR 'gender identity' OR 'gender expression' OR 'gender presentation' OR 'gender normative' OR 'gender non-conforming' OR 'gender role' OR 'gender norm' OR 'gender relation' OR 'masculinity' OR 'feminity' OR 'feminine presenting' OR 'masculine presenting' OR 'doing gender' OR 'gender mainstreaming' OR 'domestic violence' OR 'sexism' OR 'racialised' OR 'racialized' OR 'homophobic' OR 'transphobic' OR 'homophobe' OR 'transphobe' OR 'homophobia' OR 'transphobia' OR 'sexist' OR 'misogyny' OR 'heteronormativity' OR 'lgbt' OR 'lgbtqi' OR 'transgender' OR 'transgendered' OR 'transsexual' OR 'cisgender' OR 'intersex' OR 'sexual minority' OR 'sexual minorities' OR 'gender minorities' OR 'gender minority' OR 'queer' OR 'gay' OR 'lesbian' OR 'sexual orientation' OR 'men who have sex with men' OR 'women who have sex with women')

AND ('health' OR 'well-being' OR 'mortality' OR 'life expectancy' OR 'morbidity' OR 'risk' OR 'disease' OR 'physical' OR 'psychological' OR 'mental health' OR 'health inequalities' OR 'depression' OR 'anxiety' OR 'psychosis' OR 'stress' OR 'minority stress' OR 'ptsd' OR 'post-traumatic stress syndrome' OR 'health status'/exp OR 'health equity'/exp OR 'health service'/exp OR 'sexual health'/exp OR 'health behaviour'/exp OR 'health promotion'/exp OR 'health information'/exp OR 'men`s health'/exp OR 'child health'/exp OR 'women`s health'/exp) AND [humans]/lim AND [embase]/lim

## PsycINFO (n=2,361)

(("migration" OR "migrant" OR "migrants" OR "immigrant" OR "immigrants" OR “refugee” OR “refugees” OR “asylum” OR “undocumented” OR "first generation" OR "second generation" OR "ethnic minorities" OR "ethnic minority" OR “racial minority” OR “racial minorities” OR DE "refugees" OR DE “Human Migration” OR DE”Immigration”)) )

AND ( (“health” OR “well-being” OR “mortality” OR "life expectancy" OR “morbidity” OR “risk” OR “disease” OR "physical" OR "psychological" OR "mental health" OR "health inequalities" OR "depression" OR “anxiety” OR “psychosis” OR “stress” OR “minority stress” OR “PTSD” OR “post-traumatic stress syndrome” OR DE “health disparities” OR DE “health care services” OR “sexual health” OR “child’s health” OR “men’s health” OR “women’s health” OR DE “health behavior” OR DE “health promotion” OR DE “health knowledge”) )

AND ( (TI “gender” OR “gender difference” OR “gender differences” OR “gender-based” OR “gender-dependent” OR “gender violence” OR “gender bias” OR “sex bias” OR “gender relations” OR “gendered violence” OR “gender discrimination” OR “gendered discrimination” OR “gender inequalities” OR “gender inequality” OR “gender equality” OR “gender identity” OR “gender expression” OR “gender presentation” OR “gender normative” OR “gender non-conforming” OR “gender role” OR “gender norm” OR “gender relation” OR “masculinity” OR “feminity” OR “feminine presenting” OR “masculine presenting” OR “doing gender” OR “gender mainstreaming” OR “domestic violence” OR “sexism” OR ”racialised” OR “racialized” OR “homophobic” OR “transphobic” OR “homophobe” OR “transphobe” OR “homophobia” OR “transphobia” OR “sexist” OR “misogyny” OR “heteronormativity” OR “LGBT” OR “LGBTQI” OR “transgender” OR “transgendered” OR “transsexual” OR “cisgender” OR “intersex” OR “sexual minority” OR “sexual minorities” OR “gender minorities” OR “gender minority” OR “queer” OR “gay” OR “lesbian” OR “sexual orientation” OR “Men Who Have Sex With Men” OR “Women Who Have Sex With Women“) ) Population Group: Human, Source Types: Academic Journals

## CINAHL (n=940)

("migration" OR "migrant" OR "migrants" OR "immigrant" OR "immigrants" OR “refugee” OR “refugees” OR “asylum” OR “undocumented” OR "first generation" OR "second generation" OR "ethnic minorities" OR "ethnic minority" OR “racial minority” OR “racial minorities” OR MH "refugees" OR MH "emigration and immigration")

AND (“health” OR “well-being” OR “mortality” OR "life expectancy" OR “morbidity” OR “risk” OR “disease” OR "physical" OR "psychological" OR "mental health" OR "health inequalities" OR "depression" OR “anxiety” OR “psychosis” OR “stress” OR “minority stress” OR “PTSD” OR “post-traumatic stress syndrome” OR health status OR health equity OR health service OR sexual health OR child’s health OR women’s health OR men’s health OR health behaviour OR health promotion OR health information)

AND (TI “gender” OR “gender difference” OR “gender differences” OR “gender-based” OR “gender-dependent” OR “gender violence” OR “gender bias” OR “sex bias” OR “gender relations” OR “gendered violence” OR “gender discrimination” OR “gendered discrimination” OR “gender inequalities” OR “gender inequality” OR “gender equality” OR “gender identity” OR “gender expression” OR “gender presentation” OR “gender normative” OR “gender non-conforming” OR “gender role” OR “gender norm” OR “gender relation” OR “masculinity” OR “feminity” OR “feminine presenting” OR “masculine presenting” OR “doing gender” OR “gender mainstreaming” OR “domestic violence” OR “sexism” OR ”racialised” OR “racialized” OR “homophobic” OR “transphobic” OR “homophobe” OR “transphobe” OR “homophobia” OR “transphobia” OR “sexist” OR “misogyny” OR “heteronormativity” OR “LGBT” OR “LGBTQI” OR “transgender” OR “transgendered” OR “transsexual” OR “cisgender” OR “intersex” OR “sexual minority” OR “sexual minorities” OR “gender minorities” OR “gender minority” OR “queer” OR “gay” OR “lesbian” OR “sexual orientation” OR “Men Who Have Sex With Men” OR “Women Who Have Sex With Women“); **Human; Source Types: Academic Journals**

## EconLit (n=921)

("migration" OR "migrant" OR "migrants" OR "immigrant" OR "immigrants" OR “refugee” OR “refugees” OR “asylum” OR “undocumented” OR "first generation" OR "second generation" OR "ethnic minorities" OR "ethnic minority" OR “racial minority” OR “racial minorities” OR "emigration and immigration")

AND (“health” OR “well-being” OR “mortality” OR "life expectancy" OR “morbidity” OR “risk” OR “disease” OR "physical" OR "psychological" OR "mental health" OR "health inequalities" OR "depression" OR “anxiety” OR “psychosis” OR “stress” OR “minority stress” OR “PTSD” OR “post-traumatic stress syndrome” OR health status OR health equity OR health service OR sexual health OR child’s health OR women’s health OR men’s health OR health behaviour OR health promotion OR health information)

AND (TI “gender” OR “gender difference” OR “gender differences” OR “gender-based” OR “gender-dependent” OR “gender violence” OR “gender bias” OR “sex bias” OR “gender relations” OR “gendered violence” OR “gender discrimination” OR “gendered discrimination” OR “gender inequalities” OR “gender inequality” OR “gender equality” OR “gender identity” OR “gender expression” OR “gender presentation” OR “gender normative” OR “gender non-conforming” OR “gender role” OR “gender norm” OR “gender relation” OR “masculinity” OR “feminity” OR “feminine presenting” OR “masculine presenting” OR “doing gender” OR “gender mainstreaming” OR “domestic violence” OR “sexism” OR ”racialised” OR “racialized” OR “homophobic” OR “transphobic” OR “homophobe” OR “transphobe” OR “homophobia” OR “transphobia” OR “sexist” OR “misogyny” OR “heteronormativity” OR “LGBT” OR “LGBTQI” OR “transgender” OR “transgendered” OR “transsexual” OR “cisgender” OR “intersex” OR “sexual minority” OR “sexual minorities” OR “gender minorities” OR “gender minority” OR “queer” OR “gay” OR “lesbian” OR “sexual orientation” OR “Men Who Have Sex With Men” OR “Women Who Have Sex With Women“) Source Types: Academic Journals

## Cochrane Library (n=618)

(("migration" OR "migrant" OR "migrants" OR "immigrant" OR "immigrants" OR “refugee” OR “refugees” OR “asylum” OR “undocumented” OR "first generation" OR "second generation" OR "ethnic minorities" OR "ethnic minority" OR “racial minority”)

AND (“health” OR “well-being” OR “mortality” OR "life expectancy" OR “morbidity” OR “risk” OR “disease” OR "physical" OR "psychological" OR "mental health" OR "health inequalities" OR "depression" OR “anxiety” OR “psychosis” OR “stress” OR “minority stress” OR “PTSD” OR “post-traumatic stress syndrome” OR health status OR health equity OR health service OR sexual health OR child’s health OR women’s health OR men’s health OR health behaviour OR health promotion OR health information)

AND (“gender difference” OR “gender differences” OR “gender-based” OR “gender-dependent” OR “gender violence” OR “gender bias” OR “sex bias” OR “gender relations” OR “gendered violence” OR “gender discrimination” OR “gendered discrimination” OR “gender inequalities” OR “gender inequality” OR “gender equality” OR “gender identity” OR “gender expression” OR “gender presentation” OR “gender normative” OR “gender non-conforming” OR “gender role” OR “gender norm” OR “gender relation” OR “masculinity” OR “feminity” OR “feminine presenting” OR “masculine presenting” OR “doing gender” OR “gender mainstreaming” OR “domestic violence” OR “sexism” OR ”racialised” OR “racialized” OR “homophobic” OR “transphobic” OR “homophobe” OR “transphobe” OR “homophobia” OR “transphobia” OR “sexist” OR “misogyny” OR “heteronormativity” OR “LGBT” OR “LGBTQI” OR “transgender” OR “transgendered” OR “transsexual” OR “cisgender” OR “intersex” OR “sexual minority” OR “sexual minorities” OR “gender minorities” OR “gender minority” OR “queer” OR “gay” OR “lesbian” OR “sexual orientation” OR “Men Who Have Sex With Men” OR “Women Who Have Sex With Women“)) (Word variations have been searched)
