# Supplementary material for: Representation of gender in migrant health studies – a systematic review of the social epidemiological literature
Source: Int J Equity Health. 2020 Oct 14;19:181. doi: 10.1186/s12939-020-01289-y (PMC7556985; doi:10.1186/s12939-020-01289-y)
Supplement: Supplementary file 4 — Additional file 4. [file 12939_2020_1289_MOESM4_ESM.docx]

# Quality Assessment for cross-sectional and cohort studies

| **Criteria**  **Short reference** | **Q1** | **Q2** | **Q3** | **Q4** | **Q5** | **Q6** | **Q7** | **Q8** | **Q9** | **Q10** | **Q11** | **Q12** | **Q13** | **Q14** | **Quality rating** | **Comments** |
| --- | --- | --- | --- | --- | --- | --- | --- | --- | --- | --- | --- | --- | --- | --- | --- | --- |
| Arnoso et al. 2017 | 🗸 | 🞫 | NR | CD | 🞫 | 🞫 | 🞫 | 🗸 | 🗸 | NA | 🗸 | 🞫 | NA | 🗸 | Fair | 2/4: population not clear, no eligibility criteria 9/11: use of (adapted versions of) validated scales 14: only adjust for age and sex |
| Bermudez et al. 2010 | 🗸 | 🗸 | 🞫 | 🗸 | 🞫 | 🞫 | 🞫 | 🗸 | 🗸 | NA | 🗸 | 🞫 | NA | 🗸 | Fair | 2: time period missing 9: subjective, self-report measure  10: based on self-report, formula not validated |
| Bianchi et al. 2004 | 🗸 | 🗸 | NR | 🗸 | 🞫 | 🞫 | 🞫 | 🗸 | 🗸 | NA | 🗸 | 🞫 | NA | 🗸 | Fair | 2: time period missing 4: respondent-driven sampling 9: adapted version of a validated scale, self-report, subjective 11: adapted version of a validated version, self-reported |
| Bosque-Prous et al. 2015 | 🗸 | 🗸 | 🞫 | 🗸 | 🞫 | 🞫 | 🞫 | 🗸 | 🗸 | NA | 🗸 | 🗸 | NA | 🗸 | Good | 3: authors state that in some countries the response rate was very low 9: gender empowerment measure, register-based, objective 11: validated scale, self-report |
| Bruce et al. 2008 | 🗸 | 🗸 | NR | 🗸 | 🞫 | 🞫 | 🞫 | 🗸 | 🗸 | NA | 🗸 | 🞫 | NA | 🗸 | Fair | 4: description of recruitment not clear, eligibility criteria not specified, respondent-driven sampling 9/11: measures are not validated, but based on instruments and described 14: mental health status is not recognised, but is likely to be associated with risk behaviour and internalised stigma |
| Cespedes et al. 2008 | 🗸 | 🗸 | 🞫 | 🗸 | 🞫 | 🞫 | 🞫 | 🗸 | 🗸 | NA | 🗸 | 🞫 | NA | 🗸 | Good | 2: time period missing, location unspecific 3: 30% response rate 4: all recruited from same population but eligibility criteria not stated 9: subjective, self-report, not validated 11: validated instruments, still self-report 14: education, SES? |
| Choi et al. 2013 | 🗸 | 🗸 | NR | 🗸 | 🞫 | 🞫 | 🞫 | 🗸 | 🗸 | NA | 🗸 | 🞫 | NA | 🗸 | Fair | 4: chain-referral sampling methodology 9: built on validated scales, revised them after focus group input, pretested the scales (self-reported, subjective) 11: validated scale |
| Coleman et al. 2016 | 🗸 | 🗸 | NR | 🗸 | 🞫 | 🞫 | 🞫 | 🗸 | 🗸 | NA | 🗸 | 🞫 | NA | 🗸 | Fair | 8: only for gender, migration is foreign-born vs. not foreign-born 9: validated measure for gender, self-report 11: not validated, self-reported |
| DasGupta et al. 1998 | 🗸 | 🞫 | NR | NR | 🞫 | 🞫 | 🞫 | 🗸 | 🗸 | NA | 🗸 | 🞫 | NA | 🞫 | Poor | 2/4: sample recruitment not described, no eligibility criteria 9/11: very short description of measures, but validated 14: only control for sex, age and education? Not clear. Table not shown. |
| Ertl 2018 Sexual risk | 🗸 | 🗸 | NR | 🗸 | 🞫 | 🞫 | 🞫 | 🗸 | 🗸 | NA | 🗸 | 🞫 | NA | 🗸 | Fair | 2: time period missing  4: no eligibility criteria specified, convenience sample 9/11: self-report, not validated |
| Ertl 2018 Alcohol use | 🗸 | 🗸 | NR | 🗸 | 🞫 | 🞫 | 🞫 | 🗸 | 🗸 | NA | 🗸 | 🞫 | NA | 🗸 | Fair | 2: time period missing, very short description respondent-driven sampling 9: marianismo measure is subjective and self-reported, but validated 11: acculturation measure is validated |
| Fernbrant et al. 2016 | 🗸 | 🗸 | 🗸 | 🗸 | 🞫 | 🞫 | 🞫 | 🗸 | 🞫 | NA | 🗸 | 🗸 | NA | 🗸 | Good | 3: register-based 5: No. But not needed as all eligible participants were included. 9: No: the authors used the gender Equity Index for the year 2012 and used it to categorised the country of origin of women who died between 1991 and 2007. Would the 2012 GEI accurately reflect the state fo the country over those 16 years?  10: GEI is only measured once, although study covers a period from 1991-2007 --> have all countries changed in the same manner? probably not 11: register-based, death certificates --> strong exposure & outcome measure |
| Gilbert et al. 2013 | 🗸 | 🗸 | NR | 🗸 | 🞫 | 🞫 | 🞫 | 🗸 | 🗸 | NA | 🗸 | 🞫 | NA | 🗸 | Fair | 2/4: non-probability sampling, Respondent-Driven Sampling 9/11: gender & outcome measure are not validated, both subjective and self-reported |
| Gilbert et al. 2014 | 🗸 | 🗸 | NR | 🗸 | 🞫 | 🞫 | 🞫 | 🗸 | 🗸 | NA | 🗸 | 🞫 | NA | 🗸 | Fair | 2/4: non-probability sampling, Respondent-Driven Sampling 9/11: gender & outcome measure are not validated, both subjective and self-reported |
| Gonzalez et al. 2010 | 🗸 | 🗸 | 🗸 | 🗸 | 🞫 | 🞫 | 🞫 | CD | 🞫 | NA | 🞫 | 🞫 | NA | 🗸 | Poor | 2/4: convenience sample, time period is missing, as well as demographics 3: authors estimate that 10% of contacted people declined participation 9/11: description of outcome and exposure is not clear |
| Harris et al. 2005 | 🗸 | 🗸 | NR | 🗸 | 🞫 | 🞫 | 🞫 | 🗸 | 🗸 | NA | 🗸 | 🞫 | NA | 🗸 | Good | 2/4: no access to internet, cannot determine, stratified, randomised household survey  9/11: detailed description, but not validated, subjective and self-report |
| Kira et al. 2010 | 🗸 | 🗸 | 🗸 | 🗸 | 🞫 | 🞫 | 🞫 | 🗸 | 🗸 | NA | 🗸 | 🞫 | NA | 🞫 | Fair | 9/11: validated scales, self-report 14: do not adjust for demographic variables (age, social security) |
| Klein et al. 2017 | 🗸 | 🗸 | NR | 🗸 | 🞫 | 🞫 | 🞫 | 🗸 | 🗸 | NA | 🗸 | 🞫 | NA | 🗸 | Good | Random sample, validated gender & outcome measure (but self-report) |
| Kocken et al. 2006 | 🗸 | 🗸 | 🞫 | 🗸 | 🞫 | 🞫 | 🞫 | 🗸 | 🗸 | NA | 🗸 | 🞫 | NA | 🗸 | Good | 2/4: random sample (register) 3: response rate 37.8% 9/11: self-report, not validated instruments |
| Mahalingam et al. 2008 | 🗸 | 🞫 | NR | 🞫 | 🞫 | 🞫 | 🞫 | 🗸 | 🗸 | NA | 🗸 | 🞫 | NA | 🞫 | Poor | 2/4: study population not defined, recruited from different populations? Eligibility criteria missing, convenience sample 9/11: subjective, self-report but validated instruments for gender & outcome 14: Education? Social support? Acculturation? |
| Marsciano et al. 2014 | 🗸 | 🗸 | 🗸 | 🗸 | 🞫 | 🞫 | 🞫 | 🗸 | 🗸 | NA | 🗸 | 🞫 | NA | 🗸 | Good | 2/4: random sample, described elsewhere 5: cannot access the study protocol 9/11: exposure & outcome measure are subjective, self-report & not validated |
| Mireshgi et al. 2008 | 🗸 | 🗸 | NR | 🗸 | 🞫 | 🞫 | 🞫 | 🗸 | 🗸 | NA | 🗸 | 🞫 | NA | 🞫 | Fair | 2/4: convenience sampling 9/11: validated scale, self-report |
| Mizuno et al. 2012 | 🗸 | 🗸 | NR | 🗸 | 🞫 | 🞫 | 🞫 | 🗸 | 🗸 | NA | 🗸 | 🞫 | NA | 🗸 | Fair | 4: respondent-driven sampling 9/11: self-reported, not validated measurements |
| Mizuno et al. 2015 | 🗸 | 🗸 | NR | 🗸 | 🞫 | 🞫 | 🞫 | 🗸 | 🗸 | NA | 🗸 | 🞫 | NA | 🗸 | Fair | 2/4: respondent-driven sampling 9: homophobia yes/no, only a subcategory, not further specified 11: not validated, self-report but specified |
| Nieves-Lugo et al. 2019 | 🗸 | 🗸 | NR | 🗸 | 🞫 | 🞫 | 🞫 | 🗸 | 🗸 | NA | 🗸 | 🞫 | NA | 🗸 | Fair | 4: convenience sample 9/11: methods section very short, do not specify the measurement of exposure/outcome, supposedly self-reported, subjective (exposure) 14: what about other STIs? Condom use? #sex partners |
| Nivette et al. 2014 | 🗸 | 🗸 | 🗸 | 🗸 | 🞫 | 🞫 | 🞫 | 🗸 | 🗸 | NA | 🗸 | 🞫 | NA | 🗸 | Good | 2: probability sample? 9: United Nations GII - objective, reliable measure 11: teacher reported, not validated 14: only adjusted for age & SES |
| Pachankis et al. 2016 | 🗸 | 🗸 | NR | 🗸 | 🞫 | 🞫 | 🞫 | 🗸 | 🗸 | NA | 🗸 | 🗸 | NA | 🗸 | Fair | 2/4: convenience sample 9: adapted version of the Growing Up LGBT America study, self-report 11: not validated, self-report |
| Pachankis et al. 2017 | 🗸 | 🗸 | CD | 🗸 | 🞫 | 🞫 | 🞫 | 🗸 | 🗸 | NA | 🗸 | 🗸 | NA | 🗸 | Good | 3: response rates varied by the recruitment channel, minimum 10% 9: gender measure is based on national legislation --> objective data 11: largely based on recommendations of the UN's GA Special Session on HIV/AIDS and the ECDC |
| Ramirez-Valles et al. 2005 | 🗸 | 🗸 | NR | 🗸 | 🞫 | 🞫 | 🞫 | 🗸 | 🗸 | NA | 🗸 | 🗸 | NA | 🗸 | Fair | 2: time period missing 4: no eligibility criteria 9: Diaz scale, validated, self-report 11: validated scale |
| Ramirez-Valles et al. 2010 | 🗸 | 🗸 | NR | 🗸 | 🞫 | 🞫 | 🞫 | 🗸 | 🗸 | NA | 🗸 | 🞫 | NA | 🗸 | Fair | 4: description of recruitment not clear, eligibility criteria not specified, respondent-driven sampling 9/11: measures are not validated, but described in detail |
| Rhodes et al. 2008 | 🗸 | 🗸 | NR | 🗸 | 🞫 | 🞫 | 🞫 | 🗸 | 🗸 | NA | 🗸 | 🞫 | NA | 🗸 | Good | 2/4: time period missing, HOMBRES study, quasi-experimental study design, random sample |
| Rhodes et al. 2013 | 🗸 | 🗸 | NR | 🗸 | 🞫 | 🞫 | 🞫 | 🗸 | 🗸 | NA | 🗸 | 🞫 | NA | 🗸 | Fair | 2/4: time period missing, respondent-driven sampling 5: refer to the a priori defined sample size but do not specify how they calculated it. 8: internalised homonegativity yes, migrant status not analysed 9/11: very short description, but validated scales |
| Rhodes et al. 2015 | 🗸 | 🗸 | NR | 🗸 | 🞫 | 🞫 | 🞫 | 🗸 | 🗸 | NA | 🗸 | 🞫 | NA | 🗸 | Fair | 4: respondent-driven sampling 9: adapted versions of validated scales, not specified 11: not validated, self-report |
| Sabina et al. 2013 | 🗸 | 🗸 | 🞫 | 🗸 | 🞫 | 🞫 | 🞫 | 🗸 | 🗸 | NA | 🗸 | 🞫 | NA | 🗸 | Good | 2: "high density area" - demographics, location not given, but probability sample 3: minimum response rate 30.7% 4: eligibility criteria given in study protocol 9/11: validated gender and health measures (however still self-reported) 14: limited amount of covariables, education not shown |
| Sandfort et al. 2017 | 🗸 | 🗸 | NR | 🗸 | 🞫 | 🞫 | 🞫 | 🗸 | 🗸 | NA | 🗸 | 🞫 | NA | 🗸 | Fair | 2/4: convenience sample, very small sample size 9/11: self-reported, subjective, not validated |
| Stempel et al. 2017 | 🗸 | 🗸 | NR | 🗸 | 🞫 | 🞫 | 🞫 | 🗸 | 🗸 | NA | 🗸 | 🞫 | NA | 🗸 | Good | 4: combination of non-random and random sampling techniques 8: binary item on gender role ideology 9: self-reported, not validated, only one item 11: validated scale of distress among immigrants |
| Tanner et al. 2014 | 🗸 | 🗸 | NR | 🗸 | 🞫 | 🞫 | 🞫 | 🗸 | 🗸 | NA | 🗸 | 🞫 | NA | 🗸 | Fair | 2: time period not specified 4: eligibility criteria not specified, community-based participatory research 9: psychometrically validated scale, self-report 11: clearly described, no validated tool, self-report |
| Van De Vijver et al. 2007 | 🗸 | 🗸 | 🞫 | 🗸 | 🞫 | 🞫 | 🞫 | 🗸 | 🗸 | NA | 🗸 | 🞫 | NA | 🗸 | Good | 4: random sample  9/11: not validated scales, self-report, subjective, but defined in detail |
| Vanderlinden et al. 2017 | 🗸 | 🗸 | NR | 🗸 | 🞫 | 🞫 | 🞫 | 🗸 | 🗸 | NA | 🗸 | 🗸 | NA | 🗸 | Good | 2/4: random sample from the IKAROS study 8/9: Global Gender Gap score (World Economic Forum) 11: following the WHO definition for exclusive breastfeeding |
| Vaughan et al. 2014 | 🗸 | 🗸 | 🗸 | 🗸 | 🞫 | 🞫 | 🞫 | 🗸 | 🗸 | NA | 🗸 | 🞫 | NA | 🗸 | Good | See Harris 2009 for detailed information. 3: response rate over 70% 2/4: nationally representative probability sample 9: validated BSI scale 11: not validated, self-reported 14: income, SES? |
| Yoshihama et al. 2014 | 🗸 | 🗸 | 🗸 | 🗸 | 🞫 | 🞫 | 🞫 | 🗸 | 🗸 | NA | 🗸 | 🞫 | NA | 🗸 | Good | 3: 64% 4: ethnic surname-based list to draw a random sample 9/11: gender and outcome measure adapted from validated instruments (AWS, Endorsement of Male Privilige Subscale of the Revised Attitudes toward Wife Abuse scale) |
| Yoshikawa et al. 2004 | 🗸 | 🞫 | NR | 🗸 | 🞫 | 🞫 | 🞫 | 🗸 | 🗸 | NA | 🗸 | 🞫 | NA | 🗸 | Fair | 2: do not specify the location of the study 9: Diaz homophobia - validated scale, self-report 11: depressive symptoms - validated scale, HIV risk behaviour not validated, self-report |

# Quality Assessment for case control study

| **Criteria**  **Short reference** | **Q1** | **Q2** | **Q3** | **Q4** | **Q5** | **Q6** | **Q7** | **Q8** | **Q9** | **Q10** | **Q11** | **Q12** | **Quality rating** |
| --- | --- | --- | --- | --- | --- | --- | --- | --- | --- | --- | --- | --- | --- |
| Kim et al. 2017 | 🗸 | 🗸 | 🞫 | 🗸 | 🗸 | 🗸 | 🞫 | 🞫 | 🞫 | 🗸 | 🞫 | 🗸 | Fair |

**Legend**

| Yes | 🗸 |
| --- | --- |
| No | 🞫 |
| Not applicable | NA |
| Not reported | NR |
| Cannot determine | CD |

As mentioned in the Methods section, we used the quality assessment tools for cohort and cross-sectional studies and for case-control studies developed by the NHLIB to assess the quality of the studies. With regard to the first one, it is to be noted that because it is also designed for cohort studies, three questions systematically did not apply to the cross-sectional studies (Q6, Q7, Q13). However, answers “No” or “NA” to those questions were not considered as shortcomings in calculating the final score. Although we used the rating guidelines provided by the NHLIB, we slightly relaxed the conditions for granting a yes in Q2: if the authors did not give the timeframe of the study (as in the majority of included articles), we still gave a “yes” if the other elements were answered. However, we made a note in the comment box about the lack of timeframe. We also had to exercise our own judgement for the overall scoring process as is the case with most of quality assessment tools, combining answers to the questions, additional comments and discussing the overall rating of each article between reviewers.
